# *Grandi Byen*—supporting child growth and development through integrated, responsive parenting, nutrition and hygiene: study protocol for a randomized controlled trial

**DOI:** 10.1186/s12887-021-03089-x

**Published:** 2022-01-21

**Authors:** Patricia L. Kohl, Emmanuel A. Gyimah, Jenna Diaz, F. Matthew Kuhlmann, Sherlie Jean-Louis Dulience, Fithi Embaye, Derek S. Brown, Shenyang Guo, Joan L. Luby, Jennifer L. Nicholas, Jay Turner, Melissa Chapnick, Joseline Marhone Pierre, Jacques Boncy, Rony St. Fleur, Maureen M. Black, Lora L. Iannotti

**Affiliations:** 1 Brown School, Washington University in St. Louis, 1 Brookings Dr., Campus Box 1196, St. Louis, MO 63130 USA; 2 Division of Pediatric Gastroenterology, Hepatology and Nutrition, Department of Pediatrics, Washington University School of Medicine in St. Louis, 660 S. Euclid Ave., St. Louis, MO 63110 USA; 3 Division of Infectious Diseases, Washington University School of Medicine in St. Louis, 660 S. Euclid Ave., St. Louis, MO 63110 USA; 4 Department of Psychiatry, Washington University School of Medicine in St. Louis, 660 S. Euclid Ave., St. Louis, MO 63110 USA; 5 Department of Radiology, School of Medicine, Case Western Reserve University, 10900 Euclid Ave., Cleveland, OH 44106 USA; 6 McKelvey School of Engineering, Washington University in St. Louis, 1 Brookings Dr., St. Louis, MO 63130 USA; 7 Unité de Coordination du Programme National d’Alimentation et de Nutrition, Ministère de la Santé Publique et de la Population, 1, Angle Avenue Maïs Gaté et, Rue Jacques Roumain, Port-au-Prince, Haiti; 8 Laboratoire National de Santé Publique, Ministère de la Santé Publique et de la Population, 1, Angle Avenue Maïs Gaté et, Rue Jacques Roumain, Port-au-Prince, Haiti; 9 Hôpital Universitaire Justinien, Cap-Haitien, Haiti; 10 Department of Pediatrics, University of Maryland School of Medicine, 655 W. Baltimore Street, Baltimore, MD 21201 USA

**Keywords:** Stunting, Responsive parenting, Child development, Integrated parenting intervention, Nutrition

## Abstract

**Background:**

Poor child growth and development outcomes stem from complex relationships encompassing biological, behavioral, social, and environmental conditions. However, there is a dearth of research on integrated approaches targeting these interwoven factors. The *Grandi Byen* study seeks to fill this research gap through a three-arm longitudinal randomized controlled trial which will evaluate the impact of an integrated nutrition, responsive parenting, and WASH (water, sanitation and hygiene) intervention on holistic child growth and development.

**Methods:**

We will recruit 600 mother-infant dyads living in Cap-Haitien, Haiti and randomize them equally into one of the following groups: 1) standard well-baby care; 2) nutritional intervention (one egg per day for 6 months); and 3) multicomponent *Grandi Byen* intervention (responsive parenting, nutrition, WASH + one egg per day for 6 months). Primary outcomes include child growth as well as cognitive, language, motor, and social-emotional development. The study also assesses other indicators of child health (bone maturation, brain growth, diarrheal morbidity and allergies, dietary intake, nutrient biomarkers) along with responsive parenting as mediating factors influencing the primary outcomes. An economic evaluation will assess the feasibility of large-scale implementation of the interventions.

**Discussion:**

This study builds on research highlighting the importance of responsive parenting interventions on overall child health, as well as evidence demonstrating that providing an egg daily to infants during the complementary feeding period can prevent stunted growth. The multicomponent *Grandi Byen* intervention may provide evidence of synergistic or mediating effects of an egg intervention with instruction on psychoeducational parenting and WASH on child growth and development. *Grandi Byen* presents key innovations with implications for the well-being of children living in poverty globally.

**Trial registration:**

NCT04785352. Registered March 5, 2021 at https://clinicaltrials.gov/

## Background

### Introduction

Stunted growth and suboptimal developmental outcomes during a child’s early years can induce dire health consequences such as increased infection risk, susceptibility to chronic diseases, impaired cognition, and poor behavioral outcomes throughout the lifespan [1]. Worldwide, significant progress has been achieved in mitigating child malnutrition, with a reduction of nearly 56 million child stunting cases over the past two decades [2]. Despite this progress, childhood stunting and poor child development remain significant global health challenges. Recent global estimates indicate that approximately 21.3% of children under the age of five—an estimated 144 million children—have stunted growth [3]. Childhood stunting is particularly rampant among the world’s poorest and most food insecure, affecting nearly a third of children under five years of age from low-income, food-deficit countries [3]. Poor early developmental outcomes can have implications for a child’s prospects in educational attainment and their future socio-economic standing, yet in low-resource settings, an estimated 250 million children are at risk of not reaching their full developmental potential due to the impacts of stunting and poverty exposure during early childhood [4].

In resource-poor settings, it is acknowledged that impaired growth and development arise from multiple etiologies including inadequate nutrition, exposure to environmental toxins, lack of cognitive stimulation, psychological stress, and nonresponsive parenting [5, 6]. These threats to child development are further compounded by poverty. Policy interventions against poverty are essential to improving child health outcomes in the long run. However, such interventions are often complicated, and achieving anticipated impacts can take several years. Targeting proximal child health factors such as nutrition and parenting behaviors can result in more drastic and timely impacts. This is particularly important, given the urgency of widespread child malnutrition and poor neurobehavioral development globally. Addressing the multifactorial determinants of child health is key to promoting global health equity and breaking cycles of poverty. Integrated interventions targeted at child growth and development outcomes ensure the maturation of children into thriving adults who are able to contribute to the sustainable development of their communities [7, 8].

In recent times, child development experts have asserted the need to shift from single-component intervention approaches (e.g. single nutrient supplements; or fortified supplement plus WASH intervention) to transdisciplinary integrated early life interventions in order to address poverty-related factors that hinder child growth and development [7–9]. There is, however, a paucity of evidence demonstrating integrated interventions that can result in more sustained child health outcomes by simultaneously targeting biological, behavioral, social, and environmental conditions associated with child growth and development. The *Grandi Byen* (“*grow well”* in Haitian Creole) study seeks to fill this gap, thereby fulfilling the urgent imperative to address the multidimensional nature of stunted growth and development. This project builds on previous findings from our team showing significant impacts of a 6-month egg intervention on child growth and nutrient biomarkers that are associated with brain development [10, 11]. It further seeks to build evidence linking egg nutrition to psychosocial indicators of child development. The study will be conducted in Cap-Haitien, Haiti’s second largest city.

Sustainable, cost-effective, multifaceted, early childhood interventions are critically needed in low-resource and food-insecure urban settings bearing a disproportionate burden of impaired child growth and development. Similar to urban communities in the United States and around the world, poverty is a crucial determinant of impaired growth and development in Cap-Haitien and other urban settings in Haiti [12]. As of 2020, an estimated 60% of the Haitian population—about 6.3 million people—lived in poverty, and 24% lived in abject poverty [13]. Moreover, food insecurity affects nearly half of Haiti’s populace [14]. Recent data from the country’s Demographic and Health Survey indicate that stunted growth affects more than one in five Haitian children under the age of five (22%), and diarrheal disease—a leading cause of child malnutrition and mortality [15]—is highest in children ages 6 to 11 months, with a prevalence of 38% in this age group [16]. In addition to poverty, Cap-Haitien’s urban context presents common challenges to child development including population density, inadequate housing, poor water and sanitation conditions, among others.

Compared with conventional food or nutrient supplementation approaches, an integrated child development and nutrition intervention premised on Haitian social, environmental, and demographic factors along with existing parenting behaviors may provide better avenues for children to reach their growth and development potential. The *Grandi Byen* intervention taps available resources with high impact potential—willing parents invested in their child’s development, affordable animal source foods (ASF) for high quality nutrition, and WASH to mitigate urban infection risks.

### Evidence from preliminary studies

The *Grandi Byen* trial builds on the following preliminary studies from our team which have centered on the individual domains related to this research—child nutrition, parenting, child development, and infection. We build on this body of evidence to combine methods and conceptual frameworks from the different research domains to guide our multidimensional approach against the threats to child growth and development.

#### Egg and nutrition trials

The first and primary trial informing this current study is the *Lulun Project*, which was led by members of our team to assess the impact of daily egg consumption on child growth and other nutrition outcomes during the complementary feeding period. A randomized controlled trial (RCT) conducted in Pastocalle, Ecuador, findings confirmed the impact of ASFs in supporting child growth and intake of critical nutrients which are often limiting in low-resource populations. For the 6-month follow-up of children who were ages 6-9 months at baseline, the results showed that children in the egg group had an increased length-for-age Z-score (LAZ) and 47% lower prevalence of stunting compared to the control group [10]. Furthermore, analyses of biomarker parameters supported the large growth effect. The egg intervention significantly increased plasma concentrations of several amino acids as well as choline and docosahexaenoic acid (DHA) compared to the control [11]. Choline is important for production of phospholipids, cell membrane integrity, conversion into acetylcholine and sphingomyelin for brain development and function [17]. There is rapid accumulation of DHA in the brain during early childhood, and this supports neurogenesis, neurotransmission, myelination, and synaptic plasticity [18]. These biomarker findings support the role of egg nutrition in growth and, potentially, brain development and function.

Single-component interventions are known to have limited impacts on long-term growth outcomes [19]; these limitations were evident in some of our findings from follow-up and replication studies, despite the generally promising findings from the *Lulun Project*. In the *Lulun Project II* cohort study, approximately 2 years from baseline, we found evidence of growth faltering, no differences between the egg and control groups in LAZ, and significant declines in LAZ from the endline *Lulun Project* in the egg intervention group relative to the control group [20]. In Malawi, the *Mazira Project* replicated the *Lulun Project* and found that the egg intervention had limited effects on linear growth [21]. Increases in intake of proteins and multiple micronutrients were, however, observed in the egg intervention group [22]. The evidence from Malawi, in particular, demonstrates the importance of understanding contextual implications when translating evidence from one setting to another. It is implied that contextual differences between Ecuador and Malawi including dietary staples, regular consumption of other high-quality ASFs in Malawi, lower stunting prevalence in Malawi, differential infection risks, and better health-related social conditions in Ecuador may have explained the contrasting outcomes in both settings [21, 23]. Particularly, the regular consumption of fish in Malawi means that a single-component intervention with ASFs alone would have limited impacts on growth and nutrition outcomes [21]. In Haiti, however, food insecurity limits consumption of ASFs at the population level [24, 25]. Thus, an intervention incorporating ASFs may result in more positive growth outcomes in this setting. An additional contextual layer to the *Grandi Byen* study is that it recognizes urbanization as a risk factor for poor child health outcomes. Urbanization is an accelerating trend globally. Families throughout the world move to cities seeking education, healthcare, and access to markets and food systems for their children, but often face the limitations of poverty [26, 27]. This current study, distinguished from the *Lulun* and *Mazira* projects which tested the egg intervention in rural settings, may offer insight into overcoming unique urban challenges to child development.

This study further builds on the *Lulun Project* by evaluating functional child development outcomes in association with egg nutrition. While the *Mazira Project* did not show differential effects of eggs on several developmental indicators [23], our team previously observed pathways from nutrition to developmental milestones in a sample of Haitian children. We analyzed data on child development domains from a trial testing a fortified peanut butter supplement, Nutributter® [28], for growth outcomes among children ages 6-11 months living in Cap-Haitien. Our analyses showed that nutrition factors such as dietary diversity, breastfeeding frequency, and child egg consumption predicted earlier achievement of motor and phonetic language outcomes [29]. Findings from this study highlighted the complex relationship between nutrition, dietary intake, infectious disease, and child development outcomes. It also demonstrated the need to delineate the additive and synergistic effects of biological, psychosocial, and environmental factors tied to child development.

This body of evidence, therefore, indicates an imperative to understand the impacts of a multifaceted approach, encompassing nutrition with parenting and nurturing care practices, on holistic child growth and neurobehavioral development—particularly in the context of Cap-Haitien, where nutrition alone has been associated with early development milestones.

#### Grandi Byen pilot study and parenting interventions

Child development findings from the Nutributter® study led our team to conduct the pilot study for *Grandi Byen*. The study involved two phases: I) formative research on child development and parenting practices in Haiti, and II) a pilot intervention study testing an integrated parenting and egg nutrition intervention. In an attempt to drift from a nutrition-only perspective on child development, the pilot study sought to generate evidence on culturally appropriate parenting practices in Haiti in order to develop a more holistic approach against poor child development outcomes. Despite the many challenges associated with parenting among low-income families, parenting interventions can improve both parent and child outcomes. Like nutritional deficiencies, non-responsive parenting is associated with a smaller hippocampus [6, 30]. Responsive parenting has also been associated with brain, cognitive, language, and social development and can buffer the effects of early adverse exposures like poverty [31–33]. Thus, the parenting focus of the pilot intervention recognizes the overwhelming evidence demonstrating that stressors associated with chronic poverty impact parenting behaviors and child outcomes [34–36].

Phase I of the pilot study involved four focus groups with parents, caregivers, and community health workers and 14 in-depth interviews to understand parental views on child development, their perceptions about what parents need to promote their child’s development, and their willingness to participate in a group-based intervention. Parents were highly concerned about their children’s health and development. They expressed an overall strong interest in learning strategies that would help their children reach their full developmental potential [37]. In this formative research, mothers were clearly identified as the primary caregivers in Haitian homes. We also observed that when mothers and fathers were both present, the men were more vocal while women often sat silently. Based on this gender dynamic and previous evidence from similar contexts [38], we made the decision to only include mothers in the current study.

With information acquired in phase I, the team adapted the parenting intervention developed by Singla and colleagues [39]—which was found to be effective with parents in Uganda—for phase II. This intervention manual was selected because of its integrated approach and parallels to the emerging themes from communities studied in phase I. Manual text and images as well as posters and counseling cards were adapted for the Haitian context. Thirty-one mother-child pairs were recruited from communities in Cap-Haitien and randomized into intervention (n=16) and control groups (n=15). The intervention group received a 12-week training, which included content on responsive parenting, provision of ASFs, and importance of sanitation and hygiene. They also received one egg daily. Post-intervention qualitative interviews revealed mothers’ positive reception to the intervention. Overall, this pilot work demonstrated the fit and appropriateness of the interventions to mothers, as well as the feasibility of delivering it using a group format within communities in Cap-Haitien. *Grandi Byen,* therefore, also builds from this pilot study to test the integrated intervention on a larger scale.

Members of our team have led multiple trials testing the effectiveness of parenting interventions, particularly among low-income populations. These trials inform the protocol and the delivery of *Grandi Byen*. In an RCT evaluating the *Pathways Triple P* program, a 14-week parenting intervention with low-income parents in the US child welfare system, there were increases—compared to standard care—in parents’ developmentally appropriate expectations of their children, parenting efficacy resulting from skill acquisition, and improved child behavioral health outcomes. At endline, parents also reported that they enjoyed their children more (unpublished observations—P.L. Kohl, Washington University in St. Louis, MO). For *Grandi Byen,* we build on formative research findings from the pilot study, as well as knowledge from investigators who are accustomed to Haitian cultural norms, and carefully consider the cultural context to adapt this parenting intervention without making major changes to its core elements. Our experience with parenting interventions has shown that hard to reach populations can be recruited and retained through intervention and assessment completion [40–43]. The current study draws from these previous research protocols. Parents’ relationship with the intervention facilitators also matter [44]; thus, facilitators leading the *Grandi Byen* parenting intervention will be trained on parent engagement strategies. Finally, our team will draw from experiences in developing and evaluating fidelity measures to ensure that the parenting intervention is being delivered as intended [45]. Taken together, this body of work suggests that a parenting intervention can be successfully tested in low-resource communities with parents living in chronic poverty.

#### Enteric disease risk

The parenting intervention of the current study incorporates WASH education, which is known to significantly reduce the risk of diarrheal disease [46]. Evidence strongly supports the synergistic effects of malnutrition and diarrheal disease on growth and neurocognitive development in resource-constrained contexts [47–49]. There is also an increasing awareness of comorbid complications such as environmental enteropathy and cognitive impairment [50]. Therefore, efforts to quantify the effect of complementary egg feeding, in tandem with appropriate WASH practices and responsive parenting, on diarrheal diseases is warranted.

Infectious disease experts on our team have conducted research examining host factors predicting enteric pathogen risk and severity, namely for enterotoxigenic *Escherichia coli* (ETEC) [51, 52]. ETEC is a leading cause of diarrheal mortality worldwide and in Haiti [53]; however, there is minimal evidence, outside of cholera and rotavirus, characterizing enteric disease pathogens in the country, especially in community settings. Diarrheal disease is highly prevalent among Haitian children, especially those within the study’s target age group (6 to 8 months) [16]. Members of our team are leading an ongoing case-control study in Cap-Haitien, which attempts to map deficiencies in egg-related biomarkers (DHA and choline) and their links with several indicators of diarrheal sequelae. The *Grandi Byen* study affords the opportunity to expand the limited evidence on enteric pathogens in Haiti by examining increased risks of enteric disease associated with WASH access and practices. Samples collected will also provide future insights into specific pathogenic contributions to enteric diseases.

### Conceptual framework

**Fig. 1:**
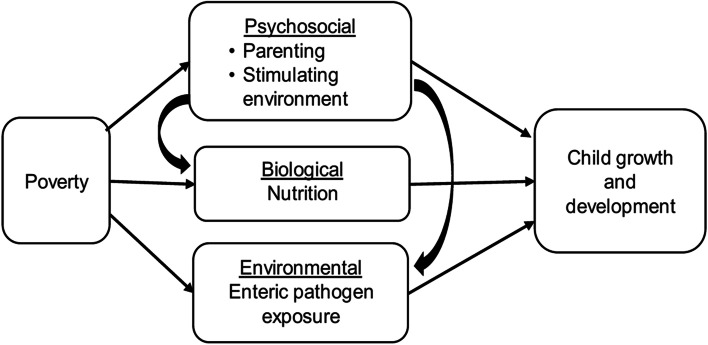
Conceptual Model of Mediating Mechanisms

The conceptual framework informing *Grandi Byen’*s rationale draws upon the work of Felner and colleagues [54], who posit an ecological mediational perspective to study the impact of poverty on human development. That is, socioeconomic disadvantage impacts development through proximal environmental experiences associated with poverty (Fig. 1). Empirical and theoretical literature suggest that psychosocial (parenting and cognitive stimulation), nutrition and environmental factors mediate the relationship between poverty and child growth and development [4]. There is a wealth of evidence emphasizing the linkages from poverty through psychosocial and biological factors to growth and developmental outcomes [4, 5, 30, 55]. However, fundamental parenting behaviors—including the provision of adequate nutrition incorporating ASFs, protection from enteric pathogen exposure through appropriate hygiene, and a cognitively stimulating environment—may attenuate the negative impact of poverty on child development. Child health interventions must target each of these mediating mechanisms.

Despite numerous recommendations from scholars over the past two decades for integrated interventions to address the multifactorial threats to child growth and development, child nutrition and health research has mainly taken the approach of testing single-component interventions. Moreover, the literature on integrated interventions remains mostly conceptual, and few rigorous trials have been conducted to determine their effectiveness [56]. Findings from the few existing integrated interventions have been promising but inconclusive [57]. A systematic review and meta-analysis assessing growth-focused early interventions in relation to effects on neurobehavioral development found that nutrition-centered interventions that improved linear growth also improved child cognition and motor development [32]. Responsive care interventions, on the other hand, were reported to have a 4 to 5 times larger effect—relative to nutrition interventions—on cognitive, language, and motor development, but limited impacts on linear growth [32]. These examples from the literature demonstrate the need for further research that assesses the synergistic impact of integrated interventions that target multiple mediating factors linked to child growth and development.

### Study aims and hypothesized pathways

Expanding on the conceptual framework (Fig. 1) and preliminary studies across the domains of child nutrition and development, parenting, and infectious disease, the *Grandi Byen* project takes a transdisciplinary approach—spanning the biological and social sciences—to study child growth and development, while also evaluating the potential for upscaling integrated child growth and development interventions in resource-constrained settings. The project’s specific aims are to: 1) demonstrate the reproducibility of egg-based interventions in reducing childhood stunting and test its impact on development; 2) investigate the incremental benefit of *Grandi Byen* compared to egg-only and standard care groups on primary outcomes of child growth and development; 3) explore pathways of intervention impacts on child growth and development by delineating the additive and synergistic effects of biological (nutrient biomarkers, bone age, and enteric disease), psychosocial (responsive parenting, maternal mental health, cognitive stimulation), and environmental (hygiene and sanitation, diet) factors; and 4) determine whether any incremental benefits associated with *Grandi Byen* when compared to an egg only intervention and standard care are favorable relative to additional costs. Table 1 describes specific hypotheses corresponding with the study’s primary aims, aims 1 and 2.

**Table 1:** Primary aims and study hypotheses

Primary study aims	Hypotheses
1. To demonstrate the reproducibility and feasibility of egg-based interventions in reducing childhood stunting and test its impact on development	Hypothesis 1: Linear growth will be increased by 0.30 LAZ in children receiving one egg per day compared to standard care
	Hypothesis 2: Children receiving the egg intervention will have better cognitive, motor and language development compared to standard care.
	Exploratory Question 1: Does an egg-based intervention affect social-emotional development?
2. To investigate the incremental benefit of *Grandi Byen* compared to egg only and standard care groups on primary outcomes of child growth and development.	Hypothesis 3: Children of mothers/caregivers receiving *Grandi Byen* will increase linear growth by 0.10 LAZ compared to the egg intervention.
	Hypothesis 4: Children of mothers/caregivers receiving *Grandi Byen* will have higher scores on child cognition, language, motor, and socio-emotional development, with an effect size of 0.36 on cognition, compared to standard care and egg nutrition groups.

The first aim allows us to replicate findings from the *Lulun Project* in a new context of Haiti and extend them to determine the effect of an egg-only intervention on developmental outcomes. Eggs alone, however, do not target the other mechanisms through which poverty impacts child growth and development. The premise of this trial is that a “bundled” intervention that combines nutrition (eggs) with a parenting intervention highlighting responsive care, ASF consumption, and improved WASH practices will act in concert to synergistically improve child growth and development over and above that of an egg intervention alone. As well as targeting additional mechanisms (e.g., responsive parenting and cognitive stimulation to promote early learning), we theorize that mothers who receive reinforcement about the importance of ASF throughout the parenting intervention will be more adherent to providing the target child with one egg per day and will continue to provide their child with diverse diets incorporating affordable ASFs post-intervention, thereby sustaining expected growth and nutrition outcomes long after the intervention ends. Aim 2 will test for the additive and synergistic effects of an egg-only nutrition intervention (arm 2) and the multicomponent intervention (arm 3) compared to standard care (arm 1).

**Fig. 2:**
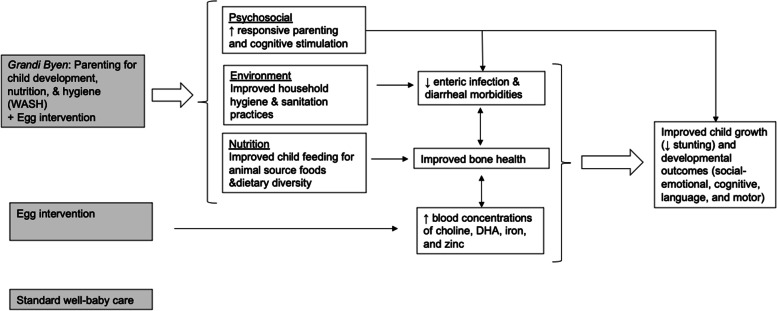
Hypothesized pathways from study conditions to outcomes

Disentangling which specific elements (psychosocial or hygiene and sanitation practices) of the intervention did most to enhance the nutrition intervention may be useful for future adaptations to other contexts. However, our emphasis will be on the effects of the bundled intervention, which is a common practice in medical research [58, 59]. In aim 3, we will explore hypothesized pathways from the interventions to growth and development impacts (Fig. 2). The *Grandi Byen* intervention is anticipated to influence parenting practices through improvements in caregiving and child stimulation, household environmental conditions for WASH, and child feeding for dietary diversity and ASF consumption, which may in turn reduce infectious disease incidence, improve bone maturation, and increase blood concentrations of critical nutrients. By contrast, the egg intervention alone may affect nutrition biomarkers, infection, or bone maturation to a lesser extent but is unlikely to strongly influence child development outcomes. Finally, children in the standard well-baby care group should show little or no impacts on growth and development.

The fourth aim highlights a major gap related to the effective translation and continuity of evidence-based programs in real-world settings by addressing economic considerations related to public health programming. We intentionally selected interventions (egg only and *Grandi Byen*) that are feasible for translation into programming and policy with preliminary evidence to support this [60]. Experts in implementation science recognize that there is limited research on cost-effectiveness of evidence-based interventions and emphasize that future research addresses this gap [61]. Multicomponent interventions are at risk for being expensive and complicated to reproduce in multiple contexts [62]. Moreover, costly interventions would likely not be sustainable in low-resource contexts [63]. The inclusion of an economic evaluation will allow us to determine whether any incremental benefits associated with *Grandi Byen* when compared to an egg-only intervention and standard well-baby care are favorable relative to additional costs, a question of efficiency. Given that millions of children globally living in poverty are not reaching their full developmental potential, there is a tremendous need for efficacious and cost-effective interventions to disrupt early childhood trajectories towards long-term deleterious outcomes.

## Methods/Design

### Overview of study design

*Grandi Byen* is a 4-year, 3-arm RCT which aims to test an integrated nutrition (one egg a day), WASH, and responsive parenting intervention on preventing child stunting and enhancing developmental outcomes. Participants will be assigned to one of the following comparison groups: 1) standard well-baby care (control); 2) nutrition intervention (one egg per day for 6 months); and 3) integrated/multicomponent *Grandi Byen* intervention (responsive parenting, nutrition, WASH + one egg per day for 6 months). This study applies an RCT design to maximize the likelihood of establishing intervention causality and to enable us to triangulate findings across multiple measures. All children in the three arms, will be followed at multiple time points from baseline to endline (12 months) on an array of measures.

Protocols for the *Grandi Byen* study have been reviewed and approved by the Bioethics Committee of the Ministry of Public Health and Population (MSPP) in Haiti (#2021-28) and the Institutional Review Board/Human Research Protection Office at Washington University in St. Louis (# 202101035). This project is funded by the Eunice Kennedy Shriver National Institute of Child Health and Development (NICHD) (R01HD098255-02) and was registered on March 5, 2021 at https://clinicaltrials.gov/ (NCT04785352).

### Sample size

The study sample will comprise 600 mother-infant dyads, with 200 eligible pairs each assigned to one of the three study conditions. Using the software Optimal Design (Version 3.01) [64], we determined that a sample of 600 participants will lend our study adequate statistical power which allows us to observe an effect from the study interventions. Using the repeated measures module of the software under the setting of a cluster randomized trial with person-level outcomes and assuming an intraclass correlation coefficient (ICC) of 0.7, we found that the study will have a statistical power of 0.80 to detect an effect size of 0.338, which is slightly above the small effect size defined by Cohen’s d. The ICC of 0.7 is typical for individual’s serial correlation found from most longitudinal studies. If the ICC is reduced to 0.3, the study can detect an even smaller effect size of 0.31.

### Setting

**Fig. 3:**
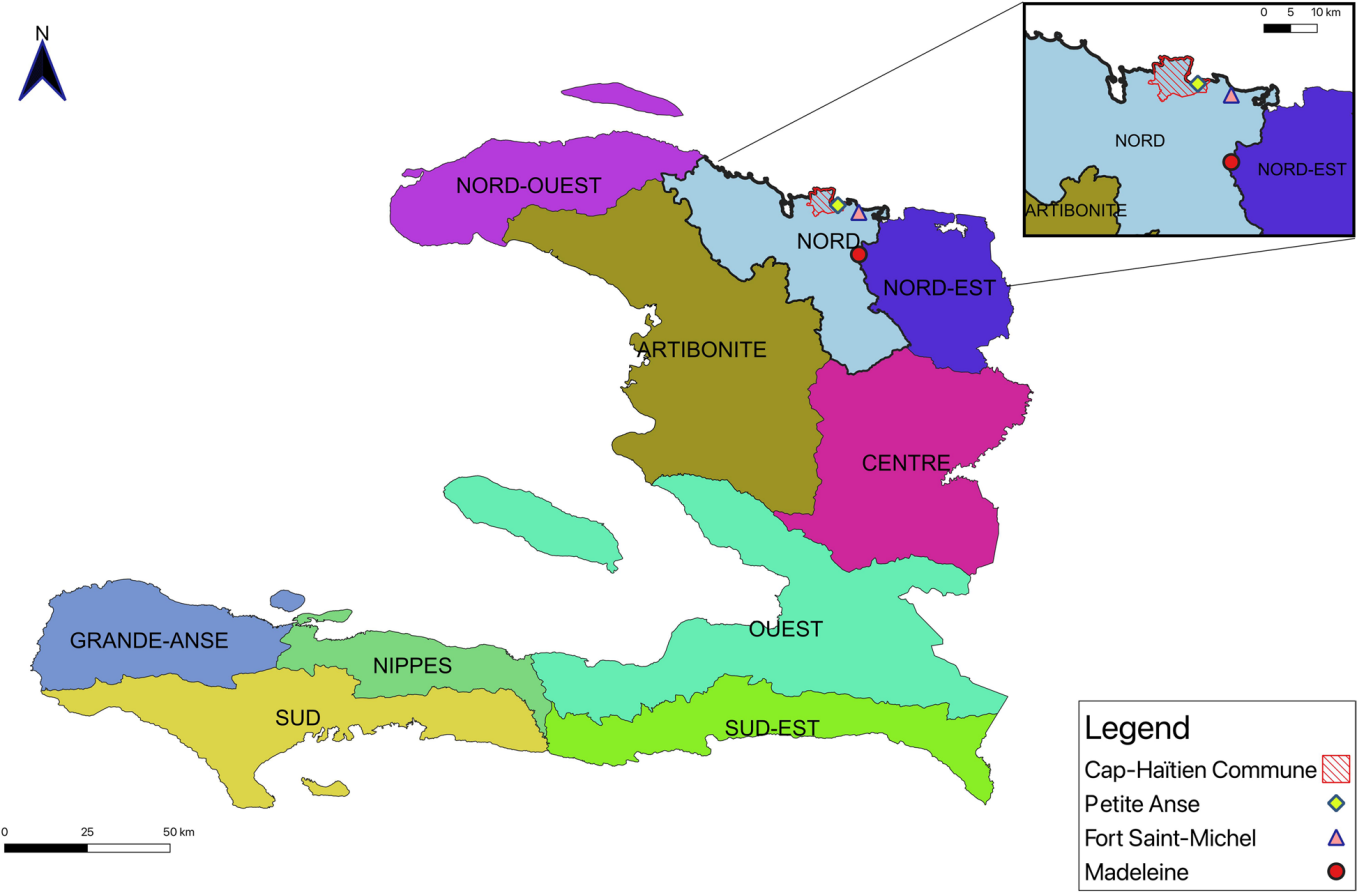
Cap-Haitien and surrounding communities. Map by E.A. Gyimah

The trial will be conducted in the urban site of Cap-Haitien and in the surrounding communities of Petite Anse, Fort Saint-Michel, and Madeleine, all located in Haiti’s Northern Department (Nord) (Fig. 3).

### Participant recruitment, eligibility, and screening

We will recruit eligible mother-infant dyads living in Cap-Haitien as well as the surrounding communities. To be eligible, mothers or caregivers must be individuals who are a minimum of 18 years old and are primary caregivers of an infant aged 6 to 8 months. Infants presenting evidence of a congenital health condition, severe disability, or severe malnourishment (weight-for-length Z-score < -3) will be excluded, because these factors may compromise child feeding and growth and can induce confounding in our results. Additionally, we will exclude children with allergies to ASFs (specifically eggs, milk, or fish) as well as those who are multi-birth infants (twin, triplet, etc.).

Prior to recruitment, potentially eligible mother-child dyads will be identified within Cap-Haitien and the three comparable communities of Petite Anse, Fort Saint-Michel, and Madeleine. The study team has conducted several studies in these communities and will draw on previously developed relationships and networks to recruit for this study in a timely manner. Community health agents, for example, regularly visit households throughout the communities and will assist in the identification and recruitment of eligible families. Additionally, medical records from the maternity and well-baby units at Fort Saint Michel clinic, and community rally posts will be used. The majority of mothers have access to cell phones, and numbers are contained in the medical records. Any information drawn from health records for mothers declining to be in the study will be destroyed.

Information sessions will be held in the communities to inform potential participants about the study. These strategies have previously enabled the team to canvas the entire catchment area and ensure all eligible mother-child pairs have equitable opportunity for entry into the study. Information sessions will be scheduled during off-work hours to better ensure all mothers have an opportunity to attend. If mothers express interest in participating, but are not able to attend the meeting, a separate enrollment date will be scheduled.

During the information sessions, the study coordinator will review and provide a full description of the study including information about the mothers’ obligations, surveys, and child nutrition and development measures. Once eligibility is established, study participants will go through the informed consent process individually. The study staff will provide the mothers with a verbal description of the study, which will explain the purpose of the study, their role in the study, and what to expect during data collection. The risks and benefits of the study will be explained to the study participants, and we will obtain written consent. Mothers will be permitted the necessary time (up until the child ages out of eligibility) to consider entry into the study to allow for careful decision-making.

Enrollment and one year of follow up are to be completed within a four-year timeframe for five respective cohorts. There will be five staggered enrollments, where 120 caregiver-infant dyads are recruited in each six-month cycle, with 40 participating dyads in each of the three study arms. All participants will be compensated for transportation costs and lost work or market time during enrollment and child assessments.

### Randomization

Using the website Randomization.com [65], we generated a list of randomization codes with a reproducible seed. These codes will be used to randomize the caregiver-infant pairs into one of the three study groups in a 1:1:1 ratio, with 200 dyads assigned to each study condition.

**Fig. 4:**
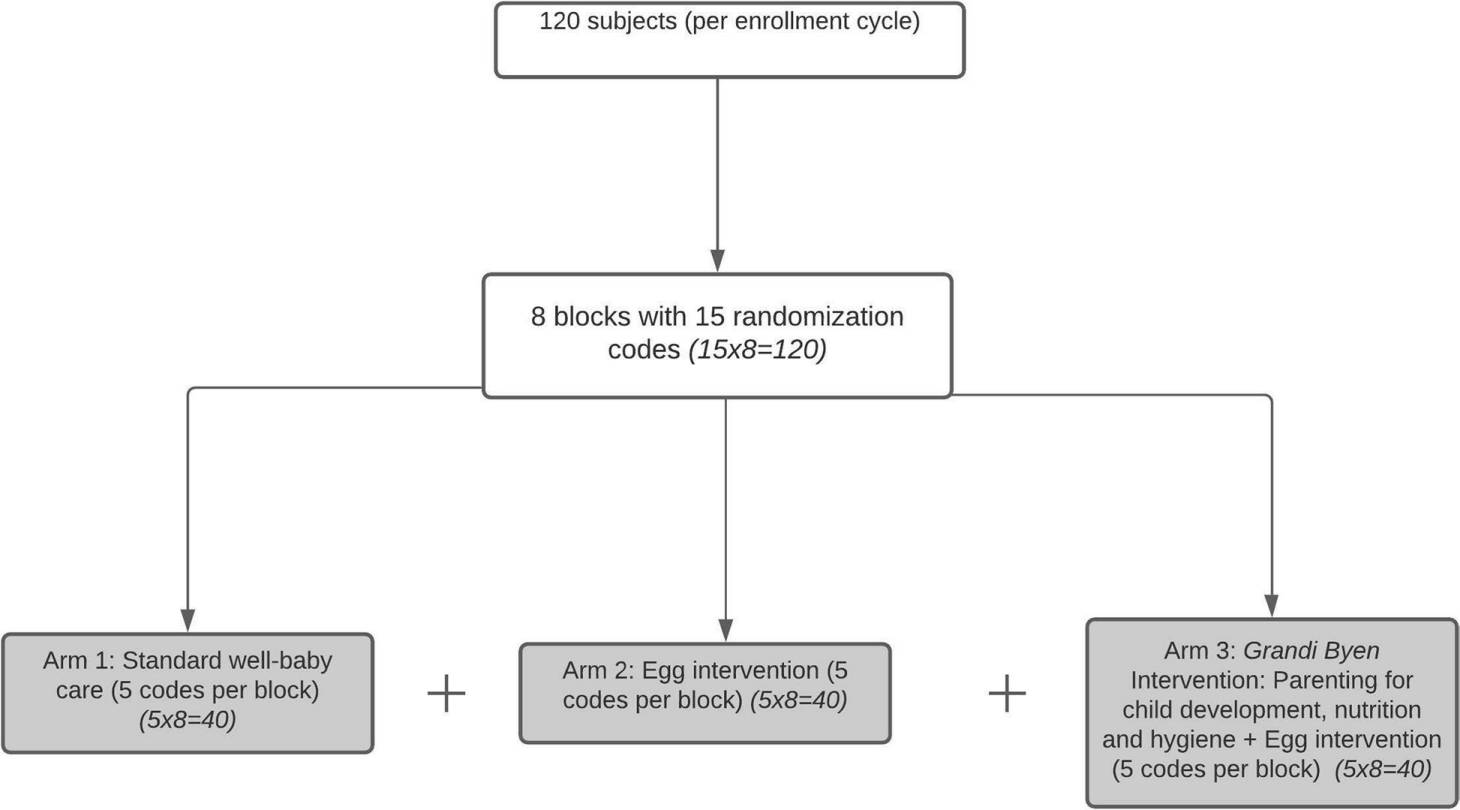
Randomization Scheme

The randomization codes are set with 120 subjects randomized into eight blocks and with each block comprising 15 subjects. Within each block, each study arm is assigned five study subjects (Fig. 4). These 120 randomization codes will be used for each of the five enrollment cycles.

Prior to the start of each enrollment cycle, a staff member, who will not be directly involved with study participants, will print 120 paper slips marked with the generated random assignment codes, seal them in opaque envelopes, and bundle them in eight blocks of 15. Each block or bundle of 15 sealed envelopes will include a random sequence of five slips each for the three study groups. Each slip will be marked with the block and card number. These numbers will be recorded in a randomization log to enable data quality checks of the group assignments.

During enrollment, the study team will explain to the mother that her child will be randomly assigned to one of the three study groups. The team will subsequently remind the mother of the differences across the three groups, invite her to pick one envelope from a basket of multiple envelopes, and explain to her that the slip inside the envelope represents the group she and her child will be assigned to.

### Description of study conditions

#### Standard well-baby care

Children in all three groups will receive standard care, which is defined by the Essential Package of Health Services from Haiti’s Ministry of Public Health and Population (MSPP) [66, 67]. This package is provided by the Haitian government at community health centers and clinics and includes a World Health Organization immunization schedule of vaccines, high-dose vitamin A supplements, and growth monitoring and promotion.

#### Egg intervention

Mother-infant dyads randomized to the two intervention groups—nutrition intervention and multicomponent *Grandi Byen* intervention—will receive a weekly ration of seven eggs for the participating index infant and additional eggs for the household. Eggs for the nutrition intervention are sourced locally from a producer who supplied eggs for the pilot study. To ensure compliance and fidelity to daily egg feeding, caregivers in these treatment groups will be given a daily log specifically designed for them to record egg consumption as well as any incidences of diarrhea during the week. When caregivers receive their weekly ration of eggs, study enumerators will collect the data recorded on these logs as part of efforts to monitor compliance. Asking caregivers to record the child’s daily egg consumption encourages self-monitoring, which is known to encourage target health behaviors [68] and may consequently promote adherence to feeding the child with an egg daily. Moreover, social marketing messages targeting mothers in the intervention groups will emphasize the importance of giving one egg per day to the index child, while addressing strategies for getting the child to consume the whole egg. Mothers will also learn about practices for hygienic handling of eggs as well as preparation techniques to optimize child nutrition.

#### Parenting intervention

Mothers randomized into the multicomponent *Grandi Byen* arm will simultaneously participate in the egg intervention and a psychoeducational parenting intervention conveying messages on nutrition, WASH practices, and responsive parenting. Originally developed and tested in Uganda [39], the group-based intervention was adapted and piloted in Cap-Haitien by the study team. The intervention is rooted in social learning theory and employs active and interactive learning strategies such as verbal instruction, modeling (vicarious experience), direct experience, and receiving feedback [69]. It is also based on cognitive, behavioral, and interpersonal therapeutic techniques that encourage shaping of knowledge, problem solving, practicing new behaviors and eliciting family social support [70, 71]. Messages are delivered and reinforced through a facilitated peer-group platform. The parenting intervention has 12 modules, emphasizing several key messages that emerged in our pilot work and are known to support healthy growth and development: maternal self-care, child development (stimulating play, interactive communication, and healthy families), complementary feeding with ASFs and continued breastfeeding, and household hygiene practices such as handwashing and use of soap. An adapted program manual guides each session. Table 2 details the 12 intervention modules for the *Grandi Byen* parenting program.

**Table 2:** *Grandi Byen* parenting intervention modules

No	Title	Session summary/empirical support	Targeted mechanism
1	Parenting for strong, healthy children	Parental aspirations; challenges encountered; introduces mothers to five principles of healthy parenting covered in *Grandi Byen*: *hygiene, food, play, conversation, self-care*. **All 5 are reinforced in every session.**	Responsive parenting
2	Strong, healthy families 1	Common family stressors that can impact child development and ways to address them. Need for positive home environment to help young children to thrive; strategies to build positive mother-infant interactions.	Responsive parenting
3	Taking care of yourself	Mothers are taught to identify and link their emotions and behaviors. Positive strategies to deal with negative emotions are introduced.	
4	Creating a stimulating environment for healthy development	Why young children need to play with objects in order to grow and learn; age-appropriate, readily available stimulating toys; mother-infant engagement	Responsive parenting
5	Talking and playing with children	New ways of talking to babies and why necessary; practice interacting with their child both verbally and non-verbally.	Responsive parenting
6	Animal source foods and diverse diets	What to feed and how much to feed infants/young children; continued breastfeeding; importance of diverse diets for young children, and why ASFs are critical in complementary feeding.	Nutrition^a^
7	Don’t forget the soap	Use of latrines and soap. Identifying barriers to handwashing and latrine use and how to overcome them.	WASH^a^
8	Positive family relationships	Strategies for positive communication and problem-solving skills for interpersonal conflict.	
9	Make play more challenging	Discusses why children need novelty and challenges. Identifies ways to make play more challenging. Identifies barriers to collecting new play things and strategies to overcome them.	Responsive parenting
10	Engaging with your children	Story-telling and other engaging activities (singing and dancing) are modeled by the facilitator and then practiced by mothers with their infants.	Responsive parenting
11	Strong, healthy families 2	This session builds upon earlier sessions focused on communication and family relationships and discusses ways to engage children’s father in play with their children. Mothers identify their own communication style and identity pros and cons of their style.	
12	Review session		

^a^Although only the primary focus of one session each, the importance of ASF and hygiene and sanitation will be reinforced weekly

Groups of 20 mother-child dyads will be formed based on location of residence in communities and will convene weekly in designated community sites (churches, schools, and community centers). Each session will last approximately one to two hours.

### Study measures

To evaluate intervention impacts, we will assess primary outcomes of child growth along with language, cognitive, motor, and social-emotional development milestones. Secondary outcomes of interest, which are also hypothesized to serve as mediating factors between the intervention and primary outcomes, include indicators for child health and nutrition (bone maturation, brain growth, respiratory or diarrheal infections, dietary intake, nutrient biomarkers), and responsive parenting based on observations of the home environment and parent-child interactions. Table 3 summarizes measures that correspond with outcomes of interest.

**Table 3:** *Grandi Byen* study outcomes

Construct	Methods	Indicator	Timepoints
**Primary outcomes**			
Child growth	Anthropometric measures of height and weight	Length-for-age Z score (LAZ) Weight-for-age Z score (WAZ) Weight-for-length Z score (WLZ)	Baseline, 3 mo, 6 mo, 9mo, 12 mo
Cognitive, Language, Motor and Social Emotional Development	Ages and Stages Questionnaires (ASQ)– Social-Emotional, Communication, Gross Motor, Fine Motor, and Problem-Solving Domains	ASQ scores	Baseline, 6 mo, 12 mo
**Secondary outcomes**			
Responsive parenting	Parenting Interactions with Children: Checklist of Observations Linked to Outcomes (PICCOLO); Home Observation Measurement of the Environment (HOME), mother-infant observation	PICCOLO: Scores in Responsiveness, Affection, and Encouragement HOME: environment subscale will be used to measure aspects of the household environment (i.e., availability of toys/items for child to play with) and responsive parenting.	Baseline, 6 mo, 12 mo
Child dietary intake	24-hour recall and food frequency questionnaire	Nutrient intakes, dietary diversity, and ASF consumption	Baseline, 6 mo, 12 mo (24-hour recall) Baseline, 3 mo, 6 mo, 9 mo, 12 mo (food frequency questionnaire)
Child nutrient biomarkers	Blood draw, mass spectrometry (LC-MS-MS, CP-OES)	Plasma concentrations of nutrient biomarkers	Baseline, 6 mo
Child bone maturation	Ultrasound imaging of hand and wrist	Bone age Z score	Baseline, 6 mo, 12 mo
Child brain growth	Ultrasound imaging of the head until anterior fontanelle closes	Dimensions of brain regions (gangliothalamic ovoid, biparietal diameter, corpus callosum, other standard measures)	Baseline, 6 mo
Child morbidities	Blood draw, immulite immunoassay methods Stool samples 2-week morbidity recall by mothers	Plasma concentrations of inflammatory biomarkers (ovomucoid and ovalbumin IgE, C-reactive Protein [CRP], and Insulin-like growth factor 1 [IGF-1]). Diarrhea, respiratory conditions, allergies	Baseline, 6 mo
Child genetics	Buccal swabs	Targeted genomic analyses	Baseline

#### Primary outcomes

*Child growth* will be evaluated using anthropometric measures. Child weight and length will be measured following the WHO’s guidelines on child growth assessments [72]. Measurements will be obtained at five time points—baseline and at 3-, 6-, 9-, and 12-month follow-ups—using the Seca Model 874 (Digital) 440 lbs x 0.1-lb. resolution and the ShorrBoard® stadiometer, respectively. All measures will be repeated at least once to ensure precision. Weight and height measures that differ by more than 0.1kg and 0.7 cm, respectively, during the second measurement will be repeated a third time. Measures will be converted to weight-for-age (WAZ), weight-for-height (WHZ), and length-for-age (LAZ) Z-scores using WHO Growth Standards [73]. Ages will be calculated using the child’s date of birth reported at baseline.

*Child development* will be assessed using the Ages & Stages Questionnaires (ASQ) at baseline and at 6- and 12-month follow-ups. The ASQ, which has been used widely for developmental screening and studies in low- and middle-income countries [74], is a parent report measure of several aspects of child development including self-regulation, affect, and social communication in very young children. The Ages & Stages Questionnaires, Third Edition (ASQ-3) will be used to assess developmental milestones along the domains of *communication* (language), *problem solving* (cognition), *fine motor*, and *gross motor* development [75], while the Ages & Stages Questionnaires: Social-Emotional, Second Edition (ASQ:SE-2) will be used to examine changes in child *social-emotional* development [76].

#### Secondary outcomes

*Responsive Parenting* will be assessed using the Parenting Interactions with Children: Checklist of Observations Linked to Outcomes (PICCOLO) [77] and the infant-toddler Home Observation Measurement of the Environment (HOME) [78]. These assessments will be completed at three time points—at baseline and at 6- and 12-month follow-ups. The validated PICCOLO assessments measure nurturing parenting behaviors, present across diverse cultures worldwide, which determine psychosocial development during a child’s early years and throughout their life course [77]. For this study, we will specifically assess parenting behaviors along the PICCOLO domains of *responsiveness*, *affection*, and *encouragement.* The environment subscale from the HOME assessment will be used in addition to the three PICCOLO domains to measure aspects of the household environment in relation to child stimulation. Assessments with these instruments will be based on a combination of mother self-report and observations by study enumerators to assess interactions between the mother and child, and the quality and quantity of stimulation in the home environment [78, 79].

Because observation within the home environment is essential to assessing these constructs, study enumerators will complete the observation items on the HOME during visits to the participants’ homes. For PICCOLO assessments, mothers will be instructed to engage with their children as they typically would for 15 minutes, and their interactions will be videotaped. Independent raters, blind to the dyad’s study condition, will review and code five-minute segments per video, randomly selected from three five-minute segments. Raters will undergo extensive training and procedures will be put in place to ensure interrater reliability.

*Child dietary intake* and levels of different *nutrient biomarkers* will be evaluated as secondary nutrition outcomes. All caregivers will undergo a 24-hour dietary recall using the standardized multi-pass interview approach [22, 80] at baseline and at 6- and 12-month follow-ups. In the first pass, caregivers will be asked to list everything the child ate and drank in the last 24 hours. In the second pass, they will report the time of consumption, ingredients, and brands of each item listed in pass one. In the third pass they will be asked to give the closest estimated amounts the child consumed using measuring cups and graduated cylinders. Culturally appropriate visual aids such as cups, dishes, and pictures of foods are also provided in order to obtain the most accurate food measurements. Lastly, the items reported are all reviewed with the mother to ensure that the history is complete. Additional information, such as current breastfeeding status will also be obtained. The goal of the 24-hour recall interview is to capture the child’s usual daily dietary intake in order to correctly estimate nutrient intakes. Mothers will therefore be asked whether the dietary intake reported represents the child’s daily usual intake. A small subset of participants will undergo repeat 24-hour recalls to assess for intra-individual variability [22]. Records from the 24-hour recall will be entered into the *NutriSurvey* software (EBISpro, Germany) [81] which will analyze and estimate nutrient intakes for each infant. Data from the 24-hour recall interviews will also enable us assess child dietary diversity as well as changes in the consumption of Animal Source Foods (ASFs). Food frequency questionnaires will also be administered at baseline and at 3-, 6-, 9- and 12-month follow-ups.

To delineate the effect of the egg intervention relative to the multicomponent intervention, we will assess different biological indicators including levels of blood biomarkers of nutritional status at baseline and 6 months post-enrollment. We will examine changes in plasma concentrations of a suite of minerals—iron, zinc, iodine, and selenium. Plasma concentrations of vitamins (choline and vitamin B12), choline-related markers (methionine and betaine), and DHA will also be assessed. A trained phlebotomist will collect 3-4 ml of venous blood from the children using trace element-free sample collection methods and processing. Blood specimens will be aliquoted and processed using sterile methods to prevent environmental contamination and subsequently stored in a -80°C freezer. Plasma mineral concentrations will be measured using inductively coupled plasma mass spectrometry (ICP-MS; PerkinElmer NexION 2000) and inductively coupled plasma optical emission spectrometry (ICP-OES: PerkinElmer Optima 7300DV). For DHA and choline-related markers, liquid chromatography-tandem mass spectrometry (LC-MS/MS) will be applied to quantify them. Vitamin B12 levels, as well markers of inflammation, will be measured using Immulite immunoassay and other methods.

Inflammatory biomarkers will be assessed in relation to caregiver reports on *child morbidities* surveys during biweekly home visits*.* Specific biomarkers of interest are ovomucoid and ovalbumin immunoglobulin E (IgE) to evaluate risk of egg allergies, C-reactive Protein (CRP) as a marker of systemic inflammation, and Insulin-like growth factor 1 (IGF-1) as an indicator of growth.

A standard two-week recall on a range of infectious illnesses will supplement CRP data. Additionally, questions to assess diarrheal severity will include the frequency of diarrhea in the children, number of sick contacts within the household, presence of blood or fever, use of anti-microbials, and requirement for additional medical care at a clinic or local provider. A similar assessment for respiratory infections including cough, rhinorrhea, fever, rash, and lethargy will be obtained through the same two-week recall survey.

Stool samples and fecal swabs will also be essential for assessments on diarrheal and infectious outcomes, future assessment of microbiome changes, and relative biomarker changes. Samples collected at baseline and at 6- and 12-month follow-ups will be associated with assessments of current or recent (past 3 days) diarrheal disease. Our pilot data coupled with national data suggests a diarrheal prevalence of 30%, supporting adequate sampling at these intervals to estimate pathogen burden (unpublished observations—F.M. Kuhlmann, Washington University School of Medicine). Stool samples will be collected from children using fecal swabs and fresh stool samples will be obtained, if available, from the child’s diaper. Should an unanticipated increase in diarrheal incidence be noted in the intervention arms, intensified efforts to identify pathogens associated with eggs (*Campylobacter* and *Salmonella*) will be pursued to determine if the egg preparations are increasing the risk of infectious diarrhea.

Ultrasound methods will be used to assess *bone maturation* at baseline and at 6- and 12-month follow-ups. *Brain growth* will also be assessed at baseline and 6 months post-enrollment using ultrasound*.* For bone maturation, ultrasound images of the child’s left hand and wrist will be obtained to assess maturity of the ossification centers. Following protocols used in our previous study in Ecuador [82], image findings will be adjusted for the child’s age and sex and indicated by the bone age Z-score (BAZ) using standards of Greulich and Pyle [83]. Additionally, brain ultrasound images will be obtained through the anterior fontanelle at baseline and 6 months. Images will assess the size of the gangliothalamic ovoid, corpus callosum, as well as ventricular diameter and extra axial space. Closure of the fontanelle will be noted, and in such cases, brain images will not be obtained. Our group conducted a feasibility study in Ecuador using a similar method [84], which will be implemented in the present study. Assessing intervention effects on child brain growth using these methods may support evidence related to DHA and choline’s role in brain development. All images will be obtained using the Mindray M7 Premium portable ultrasound with the 7L4s Linear Array Probe (Mindray North America, Mahwah, NJ).

The last biological indicator that will be evaluated is *child genetics* using buccal samples that will be obtained at the first study visit only. Buccal swab collection requires wiping the inside of the cheek with a nylon swab and placing the swab into stabilization solution. This minimally invasive and standardized technique allows for collection and preservation of genetic material and kept at room temperature for extended periods of time. Of note, interactions between host genetics, enteric pathogens, gut microbiota, and cognitive development are recently being recognized as an important component of child development [85–87]. The interpretation of the study results will almost certainly be impacted by the foresight to collect samples for retrospective targeted genomic analysis in association with the study findings.

#### Economic evaluation

*Cost data* will be obtained weekly during the parenting class for participants in arm 3 and during weekly compliance checks for participants in arms 2 and 3. For economic evaluation, research-only costs are excluded. We will measure the incremental costs of the egg (arm 2) and egg + parenting (arm 3) interventions compared to usual care (arm 1) by building participant-level cost items on time, transportation, and indirect costs into the baseline and quarterly data collection points and by measuring other intervention costs. Participants in both intervention arms may incur costs from additional time spent preparing or feeding eggs. Additionally, participants in the *Grandi Byen* arm may incur costs for time spent in the 10 sessions and for transportation. Costs to purchase, store, and deliver eggs apply to arms 2 & 3. Arm 3 also includes costs of materials, facilities for conducting the 10 sessions (community sites have an opportunity cost, even if they are provided gratis), and facilitators (for administering the intervention, 3 days of training, and transportation). Opportunity costs [88], which may differ from budgetary costs, will be measured to facilitate a social perspective.

### Statistical analyses

#### Primary and secondary outcomes

Inferential tests will be applied in the analyses for this trial. To determine differences across the three study arms, all analyses will follow an intention-to-treat framework. Moreover, all hypothesis testing will be based on a one-sided superiority paradigm because the analyses will examine whether the study interventions have beneficial impacts for the children in the study. A one-sided superiority framework is therefore legitimate, because by the theory of change for the development of the treatment program, the intervention’s positive impacts are predictable, and efforts will be made to minimize any unintended and undesirable side-effects. Specifically, the hypothesized sign for outcome variables has a positive sign, except for the child morbidities and infection, which we hypothesize to be reduced, thus indicated by a negative sign for the coefficient.

Descriptive statistics will be used to detail baseline socio-economic, demographic, and household characteristics of the study sample. Additionally, baseline and endline characteristics for the outcomes of interest will be presented in a table with means and standard deviations or medians with interquartile ranges for continuous variables and as percentages for categorical variables. Several variables represented in summary statistics will also be used as covariates in adjusted outcome analyses to ensure a convincible level of internal validity for key study variables such as the treatment effects. Additionally, covariates will be included in analyses based on the hypothesized pathways in Fig. 2 and will also be derived empirically. Empirically derived covariates will be included in analyses when a p-value < .1 results from the likelihood ratio test or the Wald test.

The primary analytic model to test primary hypotheses of the primary study aims—aims 1 and 2 (Table 1)—is the growth curve analysis using hierarchical linear modeling (HLM) or hierarchical generalized linear modeling (HGLM), depending on whether the outcome variable is a normally distributed continuous variable or a categorical variable. In the current study, the different timepoints (i.e., five waves of data collection at baseline and 3-, 6-, 9-, and 12-month follow-ups) are nested within children. These clustering effects violate the independent-observation assumption embedded in a linear model, so we will use HLM (also known as a random-effect or mixed-effect model) to control for the nesting structure of the data. In this study sample, there is only one child per parent, thus the nesting effect of children within families is absent. We therefore plan to treat mother-child pairs as one study level. However, we apply a two-level HLM model that treats time as level 1 and children as level 2.

Aim 3 examines the pathways to child growth and development by testing a series of mediational effects of secondary outcomes: responsive parenting, child dietary diversity and nutrient intakes, biomarker status, allergies, bone maturation, acute diarrhea, and respiratory morbidity. To conduct HLM or HGLM analysis for this aim, we will employ the multilevel analysis specially developed for testing mediational effects [89]. The analysis will involve three models: a full model, a reduced model, and a model using the mediator as a dependent variable. For the time-to-event outcomes (diarrhea episode, respiratory infections, and allergies in first 6 months of follow-up), we will use a proportional hazards model [90, 91].

To prevent data missingness, the field study coordinator will review the data to ensure data completeness prior to weekly data uploads to the secure Research Electronic Data Capture (REDCap) server hosted at Washington University in St. Louis [92]. The coordinator will subsequently follow up with study enumerators responsible for entering a given participant’s data to address quality issues and facilitate follow up with the study participant. Additionally, prior to all data analysis, presence, type, and number of missing observations will be explored. Logistic regression models will determine the type of missingness, whether data are missing at random (MAR), and whether the missingness is monotonic or non-monotonic. To address the possible issues with missing data, multiple imputation techniques [93] will be used to secure robust and unbiased results. Multiple imputation is highly recommended when data are MAR or missing completely at random MCAR [94, 95]. A minimum of 20 imputed files will be generated by using chained equations at the imputation stage. Outcome analysis will be performed on each imputed file, and results are aggregated by applying Rubin’s rule [93].

#### Economic evaluation

Economic assessments are specifically tied to the study’s fourth aim. To compare the efficiency of the interventions, we will conduct a cost-effectiveness analysis (CEA) to compare arm 2 to arm 1 and arm 3 to arm 2. The difference in effects (E2-E1 or E3-E2) divided by the corresponding cost difference (C2-C1 or C3-C2) constitutes the incremental cost-effectiveness ratio (ICER) [96, 97]. E1 and C1 are treated as zero for standard well-baby care. C2 and C3 are per participant costs of each respective intervention, while E2 and E3 are mean impacts for relevant study outcomes, such as LAZ or ASQ scores. Thus, multiple ICERs are reported, one for each outcome. If the ICER for 3 vs. 2 is less than the ICER for 2 vs. 1, then the costs of the more intensive intervention are offset by improvements in outcomes and arm 3 is an efficient use of resources.

For broader generalizability, we will also aggregate all study outcomes by conducting a basic cost-benefit analysis (CBA) in which non-overlapping outcome changes for arms 2 and 3 are valued in terms of local currency. Several CBA studies on nutrition in Haiti exist [98, 99] and will provide valuation estimates for most of our study outcomes, as well as a benchmark for the efficiency of this study’s intervention relative to other child health interventions in Haiti. For the CBA, B2 and B3 are the sum of all effects (E2 and E3) for the respective study arms, valued in Haitian gourdes. Benefit-cost ratios reflect efficiency (B2/C2, B3/C3), while net differences indicate how much, if any, an arm improves net welfare [(B2-B1)-(C2-C1)] and [(B3-B2)-(C3-C2)].

For both types of economic evaluation, we will conduct sensitivity analysis of the estimates using both simple 1-way (min/max) and Monte Carlo (multi-way, probabilistic) approaches to reflect statistical confidence intervals in study impacts and costs. In the CEA, costs and effects are measured in a single year, but in the CBA, benefits include a “stream” of gains (estimated into the future) and must be discounted following standard guidelines of 3-12% [96, 97, 99], a factor also included the sensitivity analysis.

## Discussion

This paper details the study protocol for the *Grandi Byen* study, a three-arm longitudinal RCT which will test the effectiveness of an integrated nutrition, responsive parenting, and WASH intervention on preventing stunted child growth and promoting neurobehavioral development. Although significant progress has been made in improving early child growth and development worldwide, disparities remain, and stunted child growth still ranks as a leading risk factor in the global burden of disease [100]. In 2012, the World Health Assembly set a target to reduce the absolute number of global child stunting cases to approximately 100 million by 2025 [101]. However, given current trends with the slow decline of child stunting prevalence, projections indicate that the global community might fail to meet this target by 27 million children [2, 101]. The urgency of this issue calls for research on transdisciplinary solutions, integrating expertise across multiple sectors, that target child nutrition as well as other proximal environmental and psychosocial factors that threaten holistic child well-being. The *Grandi Byen* study exemplifies an integrated approach offering insights into complex biological and psychosocial mechanisms, as well as feasible solutions, to confront stunted growth and development.

In conclusion, the *Grandi Byen* project adopts a “novel” conceptualization of holistic child well-being beyond growth outcomes and has the potential to provide insights into complex biological and psychosocial mechanisms that may inform evidence-based solutions against stunted growth and development. The project pools multidisciplinary concepts and frameworks from public health, social work, psychiatry, developmental psychology, infectious diseases, and radiology, merging methods to produce comprehensive perspectives and probe the underlying mechanisms of child development to enhance future interventions. This study combines key innovations implicated in healthy growth and development among children living in poverty.

## Data Availability

Data collected for this project will be made available upon appropriate ethics/human subjects research approval and reasonable request from the study's principal investigators (LLI and PLK), following the publication of the primary results from the trial. We anticipate that primary endpoint analyses will be completed within 24 months of endline data collection for the study’s final cohort.

## Abbreviations

ASF: Animal-source foods
ASQ: Ages and Stages Questionnaire
ASQ-SE: Ages and Stages Questionnaire-Social Emotional
BAZ: Bone age Z-score
CBA: Cost-benefit analysis
CEA: Cost-effectiveness analysis
CRP: C-reactive Protein
DHA: Docosahexaenoic acid
ETEC: Enterotoxigenic *Escherichia coli*
HGLM: Hierarchical generalized linear modeling
HLM: Hierarchical linear modeling
HOME: Home Observation Measurement of the Environment
ICC: Intraclass correlation coefficient
ICER: Incremental cost-effectiveness ratio
IGF-1: Insulin-like growth factor 1
IgE: immunoglobulin E
LAZ: Length-for-age Z-score
MAR: Missing at random
MCAR: Missing completely at random
MSPP: Ministry of Public Health and Population (Ministère de la Santé Publique et de la Population)
NICHD: Eunice Kennedy Shriver National Institute of Child Health and Development
NIH: National Institutes of Health
PICCOLO: Parenting Interactions with Children: Checklist of Observations Linked to Outcomes
RCT: Randomized controlled trial
WASH: Water, sanitation, and hygiene
WAZ: Weight-for-age Z-score
WHZ: Weight-for-height z-score
